# A study on post-traumatic stress disorder and post-traumatic growth among patients infected with COVID-19 in Wuhan

**DOI:** 10.3389/fpsyg.2024.1343264

**Published:** 2024-05-16

**Authors:** Jing-jing Chen, Bing Yu, Ling Yan, Xiao-xiao Sun, Qin Dai

**Affiliations:** ^1^School of Nursing, Fujian Medical University, Research Center for Nursing Humanity, Fuzhou, Fujian, China; ^2^Department of Psychology, Army Medical University, Chongqing, China; ^3^Anesthesiology Department, The 965 Hospital of the Joint Logistic, Jilin, China; ^4^The First Affiliated Hospital of Army Medical University, Chongqing, China

**Keywords:** COVID-19, physical and psychological conditions, post-traumatic growth (PTG), post-traumatic stress disorder (PTSD), nursing

## Abstract

**Objective:**

The purpose of this study is to assess the physical and psychological conditions of hospitalized patients who were infected with COVID-19 in Wuhan, China, including post-traumatic stress disorder (PTSD) and post-traumatic growth (PTG) scores and predictors.

**Methods:**

The test group consisted of 102 hospitalized patients diagnosed with COVID-19 in Wuhan between March 4, 2020 and April 5, 2020, whereas the control group comprised 168 healthy study participants. Relevant information of the study participants was obtained using online questionnaires, covering five aspects—general information, physical state, emotional state, PTSD, and PTG.

**Results:**

In Wuhan, 37.3% of COVID-19-diagnosed hospitalized patients exhibited hyper-arousal symptoms of PTSD. This percentage is significantly higher than the 13.1% observed in the healthy population. Furthermore, the prevalence of PTG among the same group of hospitalized patients stood at 77.5%, surpassing the 66.1% rate found within the healthy population. It was determined that inconsistent sleep patterns during the hospitalization phase could be indicative of heightened vulnerability to hyperarousal symptoms of PTSD in COVID-19-diagnosed hospitalized patients. The study determined that inconsistent sleep patterns during hospitalization may be a predisposition factor that makes hospitalized patients diagnosed with covid-19 more susceptible to high arousal symptoms of post-traumatic stress disorder. Conversely, COVID-19-diagnosed hospitalized patients who maintained a tranquil demeanor and exhibited positive emotional perceptions during their hospitalization displayed reduced susceptibility to these PTSD symptoms. Factors such as possession of a bachelor’s degree, history of severe acute respiratory syndrome (SARS) infection, and poor sleep patterns were identified as predictors elevating the risk of PTG. Whereas, a sentiment of happiness and consistent positive emotional perception during hospitalization were predictors of PTG. Intriguingly, a direct correlation was established between hyper-arousal symptoms of PTSD and PTG.

**Conclusion:**

Although the outbreak of COVID-19 has badly affected the physical and psychological well-being of patients, it has greatly enhanced their PTG.

## 1 Introduction

On January 30, 2020, the World Health Organization (WHO) recognized the COVID-19 outbreak as a public health emergency of an international magnitude (Li et al., 2020; Wang et al., 2020). By August 9, 2020, there were 19,462,112 globally confirmed COVID-19 cases.

Undoubtedly, COVID-19 represents a profound traumatic event, eliciting acute traumatic responses amongst patients. Numerous patients manifested various physical and psychological symptoms during the pandemic, notably sleep disturbances, anxiety, and depression (Hong et al., 2009; Lee et al., 2018). In the preliminary epicenter of the outbreak, Wuhan, in China, mandatory isolation was imposed on patients diagnosed with COVID-19. The ambiguity and apprehension associated with this novel virus invariably resulted in enhanced psychological strain and emotional turbulence among these patients, culminating, in grave instances, into post-traumatic stress disorder (PTSD).

PTSD represents a multifaceted disorder, commonly precipitating pronounced psychological distress and impeding normal social interactions. Compared to those with standard traumatic responses, patients suffering from PTSD exhibit intensified conditions marked by intrusive thoughts, evasive behavior, emotional volatility, feelings of detachment, and heightened threat alertness (Sareen et al., 2005). However, PTSD does not invariably follow traumatic events. Merely a minuscule fraction of those undergoing traumatic experiences eventually manifest PTSD. Sayed et al. stratified PTSD risk factors into pre-trauma, pri-trauma, and post-trauma phases (Sayed et al., 2015). Drawing from extant literature, our study highlighted pre-trauma predictors linked to PTSD, encompassing gender, age, educational background, and antecedent trauma exposure (Ozer et al., 2003; Kessler et al., 2005; Barnett et al., 2006; McGrath et al., 2015). Pri-trauma predictors associated with PTSD predominantly focus on the cognitive and biological trauma processing of the patients, their physiological stress response, trauma severity, and subjective emotional responses (Carlier et al., 1997; Breslau et al., 2005). Post-trauma PTSD involves psychological and societal dimensions, such as optimism, cognitive adaptability, proactive coping mechanisms, and the degree of social support.

While the COVID-19 outbreak undeniably impinged on the physical and mental health of its victims, it might paradoxically engender positive ramifications. As empirical studies suggest, traumatic incidents can catalyze post-traumatic growth (PTG) among affected patients (Arpawong et al., 2013). PTG denotes the beneficial psychological changes experienced by individuals post traumatic incidents (Tedeschi and Calhoun, 1996). Contemporary academic discourse, pivoting toward positive psychology, increasingly emphasizes the benefits individuals accrue from adversities. PTG, a prevalent aftermath of traumatic events, often leads to more pervasive positive adjustments than its maladaptive counterparts (Tedeschi and Calhoun, 2004). Recent studies demonstrated that PTG predictors exhibit significant overlaps with those of PTSD (Dekel et al., 2011; Jin et al., 2014; Tomita et al., 2017). Another large-sample study underscored a weak positive correlation between PTSD and PTG (Klosky et al., 2014).

Currently, comprehensive insights into the psychological ramifications of COVID-19 among hospitalized patients in Wuhan remain elusive. This study endeavors to elucidate the physical and mental status of such patients, concentrating on the quantifiable metrics and predictors of PTSD and PTG.

## 2 Study participants and methods

### 2.1 Study participants

The present study was of a cross-sectional design. A convenience sampling method was used. This study encompassed a sample of 102 hospitalized patients from Wuhan City, diagnosed with COVID-19 between March 4, 2020, and April 5, 2020, forming the test group. The criteria for inclusion were as follows: (1) Participants willingly engaged in the study and provided their signed informed consent. (2) Participants satisfied the COVID-19 diagnostic criteria (Diagnosis and treatment, 2020). (3) between the 3rd and 10th day of hospitalization. (4) Participants demonstrated the capability to read and exhibited a reasonable comprehension level. (5) Participants had the proficiency to complete the online questionnaires, either directly through electronic devices or with external assistance.

A control group was constituted, comprising 168 healthy individuals from WeChat (a widely used social application in China with over 1 billion active users). Their inclusion criteria comprised: (1) Participants no satisfied the COVID-19 diagnostic criteria (Diagnosis and treatment, 2020). (2) Participants provided an informed consent; (3) Participants demonstrated the capability to read and exhibited a reasonable comprehension level. (4) Participants had the proficiency to complete the online questionnaires.

Patients diagnosed with COVID-19 were recruited from Huoshenshan Hospital and Taikang Hospital, which were the largest hospitals admitting COVID-19 inpatients in Wuhan during the COVID-19 pandemic. The distribution of patients’ age, gender, and severity of disease was consistent with the overall data trend published by the National Health Commission of the People’s Republic of China. The age of healthy individuals ranged from 18 to 89 years and covered a wide range of age groups. There was no difference in gender distribution among healthy individuals (including 88 males and 80 females). In addition, healthy individuals reported all types of occupations, including farmers, soldiers, students, doctors, nurses, freelancers, police, retired workers, civil servants, sales, teachers, and others. Thus, the current sample is representative of the whole population.

### 2.2 Survey tools

In the midst of the critical epidemic response phase in Wuhan, we designed a questionnaire considering the operational feasibility and the circumstances of isolated, hospitalized patients diagnosed with COVID-19. This included taking into account their disease severity, emotional state, stress responses, and the acceptable duration for responding. Instead of adopting internationally standardized psychological test questionnaires with an exhaustive list of questions, we utilized a tailored assessment and interview questionnaire focusing on the psychological health of patients with the novel coronavirus. This tool was meticulously crafted through the concerted efforts of Professor Dai Qin’s team from the Psychological Survey and Research Division of the Army Medical University. Its development drew upon existing validated questionnaires from both domestic and international sources. Strategies such as literature review, expert consultations, and brainstorming sessions were employed. Additionally, we integrated insights from contemporary international and Chinese literature and aligned with the “Diagnosis and Treatment Scheme for Novel Coronavirus-Infected Pneumonia (Trial 5th Edition)” (Diagnosis and treatment, 2020).

Our questionnaire encompasses four domains: Pre-traumatic Information, Pri-traumatic Information, and hyper-arousal symptom of PTSD and PTG.

#### 2.2.1 Pre-traumatic information

This section captures data such as gender, age, marital status, educational level, and history of SARS infection.

#### 2.2.2 Pri-trauma information

This section was collected from two dimensions: Physical State and Emotional State.

Physical State: Here, participants provide details about their daily routines, dietary patterns, sleep quality, and disease severity. The Physical State was rated using a “yes” or “no” response. Cronbach’s alpha coefficient for Physical State was 0.617, and the KMO coefficient was 0.702, accounting for 56.2% of the total variance.

Emotional State: This part assesses emotional fluctuations across six dimensions—serenity, anxiety, anger, melancholy, fear, and happiness. The degree of emotional reaction was examined using a five-point Likert scale (how serene [anxious, angry, fearful, melancholy, and happy] you feel following your hospitalization for COVID-19): 1, no; 2, mild; 3, moderate; 4, severe; 5, extreme. The Cronbach’s alpha was 0.82, and the KMO coefficient was 0.797, accounting for 64.9% of the total variance.

#### 2.2.3 PTSD

We utilized the PTSD Symptom Checklist, a concise tool designed to gauge post-traumatic stress symptoms and promptly identify individuals predisposed to PTSD (Conybeare et al., 2012). The 17-item checklist allows respondents to rate their symptoms on a scale from 1 (indicating no specific symptom) to 5 (indicating an extremely severe symptom). Higher aggregate scores denote an elevated propensity for PTSD. This checklist has three dimensions: populations re-experiencing symptoms, populations with persistent avoidance/numbing symptoms, and populations with hyper-arousal symptoms. Our survey primarily targeted the PTSD assessment for populations with hyperarousal symptoms. The total score was calculated by summing the scores of each item (items 13–17); any item with a score ≥ 3 or any 2/5 hyper-arousal items among items 13–17 were considered positive symptoms. Cronbach’s alpha coefficient for hyper-arousal symptoms was 0.843.

#### 2.2.4 PTG

We deployed the PTG Scale, which is used to measure the beneficial psychological improvements of persons who have endured severely traumatic life events or situations (Tedeschi and Calhoun, 1996). Cronbach’s alpha coefficient for PTG was 0.90. This 21-item tool has five distinct dimensions: personal strength, relationships with others, newfound possibilities, spiritual transformation, and life appreciation. Responses range from 0 (no experienced change post-trauma) to 5 (significant post-traumatic change). PTG total scores vary from 0 to 105. Elevated aggregate scores indicate enhanced PTG levels. A benchmark score of 63 delineated the threshold for PTG presence.

### 2.3 Survey methods

We surveyed both hospitalized patients diagnosed with COVID-19 (between the 3rd and 10th day of hospitalization) and healthy study participants in Wuhan from 4th March, 2020 to 5th April, 2020, approximately the first pandemic peak of the COVID-19 outbreak in Wuhan. Data collection was facilitated through an online platform.^1^ Initially, we signed up for the Wenjuanxing platform and subsequently imported the questionnaire content into it. This process enabled us to obtain a link to the questionnaire. We then disseminated this link to the study participants’ mobile phones through WeChat, facilitating timely completion of the survey. Before the test began, hospitalized patients diagnosed with COVID-19 and healthy study participants were told the purpose of the measurements and read the informed consent to understand that they could refuse to answer or quit answering at any time. To eliminate duplicate submissions, this study limited each IP address to only one submission. By the end of the study, a total of 102 patients diagnosed with COVID-19 were recruited. All submissions from the test group were valid, yielding a 100% effective response rate. From the healthy study participants, 184 questionnaires were collected, of which 168 were deemed valid. This translated to an effective response rate of 91.3% for the control group.

### 2.4 Statistical methods

Data derived from the survey was subjected to statistical analysis using the SPSS 22.0 statistical software. A series of tests, including the two independent samples t-test, rank sum test, and chi-squared test, were applied to examine the general characteristics, physical and psychological states, post-traumatic stress reactions, and PTG levels of both the test group and the control group in Wuhan. A stratified regression analysis was specifically utilized to analyze the predictors of PTSD and PTG among the COVID-19 patient group.

## 3 Results

### 3.1 Comparison of the demographics, and physical and emotional states of hospitalized patients diagnosed with COVID-19 and healthy study participants in Wuhan

In the Pre-traumatic Information analysis, there was no statistically significant difference in gender between the test and the control groups (Table 1). Conversely, age, marital status and education level manifested statistically significant differences with a significance level of p < 0.05. In the Pre-traumatic Information analysis, with respect to Physical State, the prevalence of irregular daily routine was found to be 49.0% in patients diagnosed with COVID-19 compared to 29.2% in the control group, demonstrating a significant difference. Moreover, the incidence of poor sleep quality was registered at 43.1% for patients diagnosed with COVID-19 as opposed to 18.5% for the control group, with the difference being statistically significant at p < 0.05. From the Emotional State perspective, the proportion of individuals reporting a serene disposition amounted to 69.6% for patients diagnosed with COVID-19 and 87.5% for the control group, presenting a significant difference at p < 0.05. A detailed comparison of these variables between the groups can be found in Table 1.

**Table 1:** Comparison of the demographics, and physical and emotional states of hospitalized patients diagnosed with COVID-19 and healthy study participants in Wuhan.

	Parameter	Patients (*n* = 102)	Control group (*n* = 168)	*χ* ^2^	*p*
Pre-traumatic Information	Gender			0.287	0.592
	Male	50 (49.0%)	88 (52.4%)		
	Female	52 (51.0%)	80 (47.6%)		
	Age			29.881	<0.001
	<60	85 (83.3%)	168 (100.0%)		
	≥60	17 (16.7%)	0 (0.0%)		
	Marital status			10.433	0.001
	Married	77 (75.5%)	94 (56.0%)		
	Unmarried and others	25 (24.5%)	74 (44.0%)		
	Education level			40.186	<0.001
	Academic junior secondary school or below	27 (26.5%)	9 (5.4%)		
	Academic senior secondary school	32 (31.4%)	28 (16.7%)		
	Three-year junior college and undergraduate	40 (39.2%)	119 (70.8%)		
	Graduate or above	3 (2.9%)	12 (7.1%)		
Pri-traumatic Information	irregular daily routine			9.776	0.002
	Yes	50 (49.0%)	49 (29.2%)		
	No	55 (53.9%)	119 (70.8%)		
	Disturbances in eating			2.217	0.137
	Yes	12 (11.8%)	11 (6.5%)		
	No	90 (88.2%)	157 (93.5%)		
	Poor sleep quality			19.277	<0.001
	Yes	44 (43.1%)	31 (18.5%)		
	No	58 (56.9%)	137 (81.5%)		
	Serenity			13.066	<0.001
	Yes	71 (69.6%)	147 (87.5%)		
	No	31 (30.4%)	21 (12.5%)		
	Anxiety			2.321	0.128
	Yes	19 (18.6%)	20 (11.9%)		
	No	83 (81.4%)	148 (88.1%)		
	Anger			0.721	0.396
	Yes	2 (2.0%)	8 (4.8%)		
	No	100 (98.0%)	160 (95.2%)		
	Melancholy			0.454	0.501
	Yes	11 (10.8%)	14 (8.3%)		
	No	91 (89.2%)	154 (91.7%)		
	Fear			3.337	0.068
	Yes	9 (8.8%)	6 (3.6%)		
	No	93 (91.2%)	162 (96.4%)		
	Happiness			0.293	0.588
	Yes	19 (18.6%)	27 (16.1%)		
	No	83 (81.4%)	141 (83.9%)		

### 3.2 Comparison of hyper-arousal symptoms of PTSD and PTG between hospitalized patients with COVID-19 and healthy study participants in Wuhan

Within Wuhan, the prevalence of hyperarousal symptoms of PTSD was observed to be 37.3% among hospitalized patients diagnosed with COVID-19 compared to 13.1% in the healthy population (Table 2). Notably, this prevalence was significantly higher in the test group, as evidenced by a significance level of p < 0.05, as detailed in Table 2. Furthermore, the incidence of PTG was recorded at 77.5% for hospitalized patients with COVID-19, in contrast to 66.1% among the healthy group within the city. Again, this rate was markedly higher in the test group, with a significant difference at p < 0.05, as displayed in Table 2.

**Table 2:** Comparison of hyper-arousal symptoms of PTSD and PTG between hospitalized patients with COVID-19 and healthy study participants in Wuhan.

	Patients (*n* = 102)	Control group (*n* = 168)	*χ* ^2^ */Z*	*p*
Hyper-arousal symptoms of PTSD				
Positive	38 (37.3%)	22 (13.1%)	21.433	<0.001
Negative	64 (62.7%)	146 (86.9%)		
Total score	9 (7, 12)	6 (5, 9)	−5.891	<0.001
Item 13 Difficult falling asleep or easily waking up	2 (2, 3)	1 (1, 2)	−8.092	<0.001
Item 14 Prone to anger or outbursts of anger	1 (1, 2)	1 (1, 2)	−1.905	0.057
Item 15 Feel difficult to concentrate attention	2 (1, 2)	1 (1, 2)	−3.420	0.001
Item 16 Heightened vigilance or wariness	2 (1, 3)	1 (1, 2)	−3.031	0.002
Item 17 Excessive sensitivity and susceptibility and easily being startled	1 (1, 2)	1 (1, 1.75)	−4.150	<0.001
Post-traumatic growth (PTG)				
Positive	79 (77.5%)	111 (66.1%)	3.942	0.047
Negative	23 (22.5%)	57 (33.9%)		
Total score	83.5 (65, 96)	73.5 (56, 93)	−2.488	0.013
Interpersonal relationship	23.5 (18, 29)	21 (15, 26.75)	−2.932	0.003
New possibilities	11 (8, 12)	9 (7, 13)	−1.797	0.072
Personal strength	20 (15, 24)	18 (13, 23)	−2.030	0.042
Mental changes	8 (6, 10)	7 (5, 9.75)	−1.666	0.096
Appreciation of life	20.5 (16.75, 25)	18.5 (14.25, 23)	−2.685	0.007

### 3.3 Characteristic of the scores of hyperarousal symptoms of PTSD and PTG scores in hospitalized patients with COVID-19 in Wuhan

In Wuhan, hospitalized patients diagnosed with COVID-19 manifested significant differences in the cumulative score reflecting hyper-arousal symptoms of PTSD (Table 3). These differences were linked to physical factors, specifically, the regularity of their daily routines, the quality of their sleep, and the degree of disease from COVID-9. Emotional parameters, such as sensations of serenity, anxiety, melancholy, and fear, were also influential (p < 0.05). Furthermore, patients characterized by irregular daily routines, suboptimal sleep quality, severe illness, anxiety, melancholy, and fear registered elevated scores for hyper-arousal symptoms of PTSD. Whereas patients who portrayed a sense of serenity exhibited lower scores, as displayed in Table 3.

**Table 3:** Characteristic of the scores of hyperarousal symptoms of PTSD and PTG scores in hospitalized patients with COVID-19 in Wuhan.

Parameter	N	The total score of symptoms of PTSD at highly-aroused status	*t/F/Z*	*p*	The total score of PTG	*t/F/Z*	p
Pre-traumatic information							
Gender			−0.205	0.837*		−0.253	0.801
Male	50	9 (7, 12.25)			80.52 ± 23.002		
Female	52	9 (7, 12)			81.60 ± 19.857		
Age			−0.433	0.665*		0.386	0.700
<60	85	9 (7, 12)			81.44 ± 21.216		
≥60	17	8 (6.5, 13.5)			79.24 ± 22.615		
Marital status			−0.008	0.994*		0.179	0.858
Married	77	9 (7, 12)			81.29 ± 20.841		
Unmarried and others	25	9 (7, 12.25)			80.40 ± 23.308		
Education level			1.166	0.327		2.202	0.093
Academic junior secondary school or below	27	10.78 ± 3.796			84.96 ± 19.924		
Academic senior secondary school	32	9.59 ± 2.861			83.66 ± 21.435		
Three-year junior college and undergraduate	40	9.28 ± 3.351			75.10 ± 21.401		
Graduate or above	3	9.33 ± 3.215			98.00 ± 19.079		
History of SARS infection			−0.639	0.523**		2.456	0.017
Yes	23	9 (8.12)			77.43 ± 18.735		
No	46	9 (7, 12.25)			88.80 ± 17.828		
Pri-traumatic Information							
Irregular daily routine			−4.926	<0.001		0.931	0.354
Yes	49	12 (8, 14)			79.02 ± 22.141		
No	53	7 (6, 10)			82.96 ± 20.632		
Disturbances in eating			−0.214	0.831*		−0.104	0.917*
Yes	12	8.5 (7.25, 12.75)			82.5 (76.75, 95.25)		
No	90	9 (7, 12)			84 (65, 96.25)		
Disturbances in sleeping			−3.720	<0.001		0.729	0.468
Yes	44	12 (8, 14)			79.30 ± 21.776		
No	58	8.5 (6, 10.25)			82.41 ± 21.121		
Illness degree			−2.202	0.028		−0.045	0.964
Mild illness	90	9 (7, 12)			81.33 ± 22.500		
Severe illness	12	11 (9.25, 14.75)			81.03 ± 21.330		
Serenity			4.188	<0.001		−0.242	0.809
Yes	71	8 (7, 11)			81.41 ± 22.067		
No	31	12 (10, 15)			80.29 ± 19.959		
Anxiety			−2.962	0.003*		−0.242	0.809
Yes	19	13 (9, 15)			83.79 ± 18.305		
No	83	9 (7, 12)			80.45 ± 22.047		
Anger			−0.522	0.603		0.338	0.736
Yes	2	11.00 ± 1.414			76.00 ± 11.314		
No	100	9.75 ± 3.365			81.17 ± 21.528		
Melancholy			−2.742	0.006*		−0.242	0.810
Yes	11	13 (12, 15)			82.55 ± 20.245		
No	91	9 (7, 12)			80.89 ± 21.586		
Fear			−2.513	0.012*		−0.888	0.377
Yes	9	14 (9, 15.5)			87.11 ± 26.690		
No	93	9 (7, 12)			80.48 ± 20.852		
Happiness			−1.490	0.136*		−2.358	0.020
Yes	19	8 (6, 11)			91.26 ± 18.900		
No	83	9 (7, 13)			78.73 ± 21.300		
Positive results of hyper-arousal symptoms of PTSD			−8.289	<0.001		−1.342	0.183
Yes	38	13 (12, 15)			84.74 ± 20.460		
No	64	7.5 (6, 9)			78.89 ± 21.734		
PTG			−0.796	0.426*		−7.276	<0.001*
<63	23	9 (7, 12)			52 (48, 57)		
≥63	79	9 (7, 13)			90 (78, 103)		

Additionally, statistically significant differences were observed in the aggregate score of PTG among hospitalized patients diagnosed with COVID-19 in Wuhan when stratified by Pre-traumatic Information attributes, in particular, those who had a history of severe acute respiratory syndrome (SARS) infection. Emotional states dimension of Pri-traumatic Information, such as the presence of happiness, also influenced these scores (p < 0.05). More specifically, patients recounting feelings of happiness in their emotional experiences demonstrated higher PTG scores. In contrast, those with a prior history of SARS reflected lower PTG scores, as displayed in Table 3.

### 3.4 Analysis of predictors of PTSD in hospitalized patients diagnosed with COVID-19 in Wuhan

**Figure 1:**
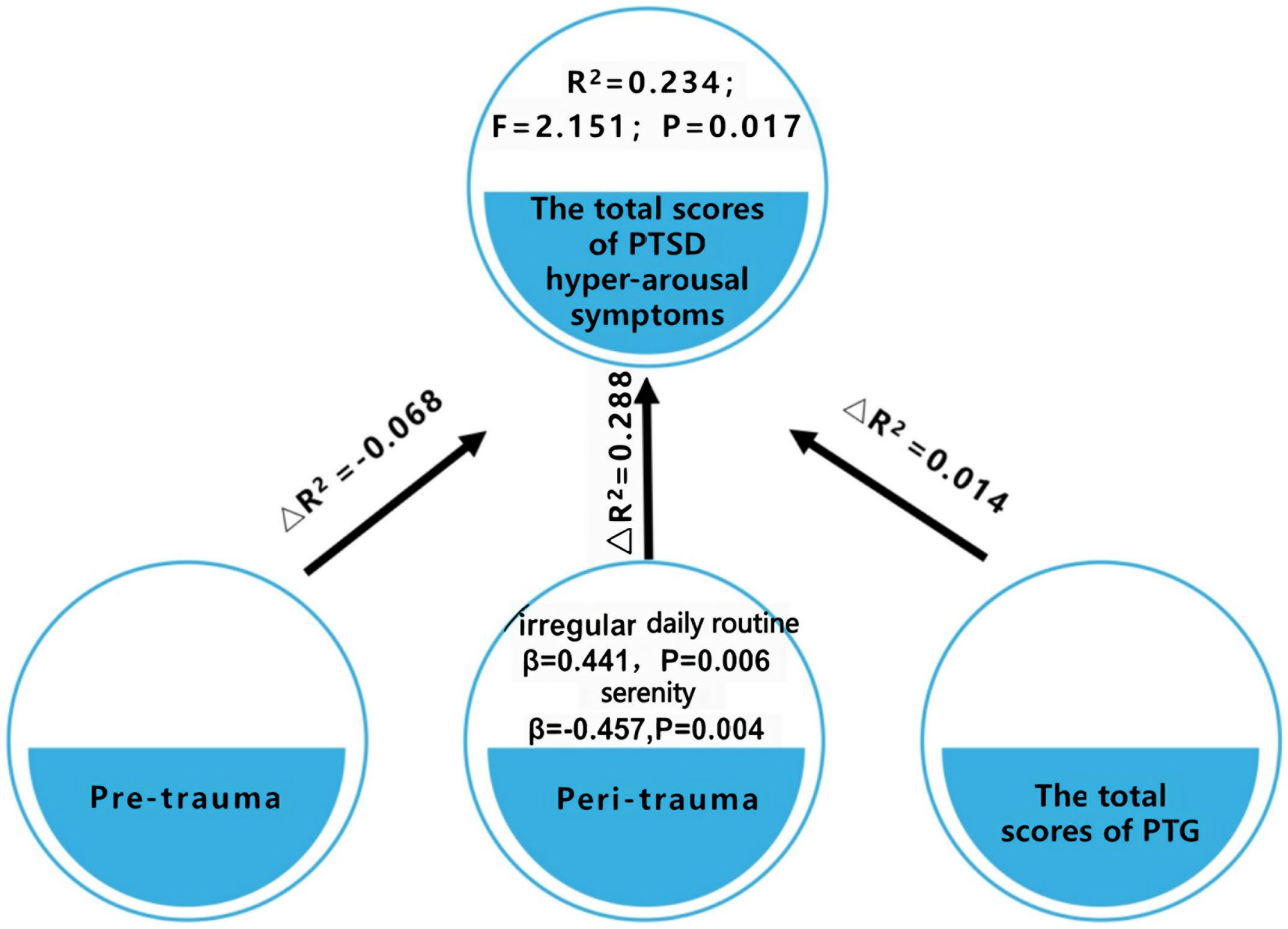
Evaluation of PTSD risk factors in patients diagnosed with COVID-19 in Wuhan.

A stratified regression analysis was performed, featuring three tiers of stratification, with the aggregate score of hyper-arousal symptoms of PTSD serving as the dependent variable (Table 4 and Figure 1). The factors considered potentially influential on the hyper-arousal symptoms of PTSD score were categorized into these three stratifications. Specifically, the initial stratification captured pre-trauma predictors, which included gender, age, marital status, educational level, and a history of SARS infection. The subsequent stratification incorporated variables encountered during the traumatic phase, namely daily routine, dietary patterns, sleep quality, disease severity, as well as emotional indicators such as serenity, anxiety, anger, melancholy, fear, and happiness. The concluding stratification was solely centered around the aggregate PTG score. Based on the results derived from this analysis, two salient variables—irregular daily routine and a sense of serenity—were integrated into the regression equation. Within this context: The pre-trauma variables were responsible for elucidating 6.8% of the variability linked to hyper-arousal symptoms of PTSD for patients diagnosed with COVID-19 in Wuhan (R^2^ = −0.068). The peri-traumatic variables elucidated 28.8% of the variability (△R^2^ = 0.288). The total PTG score accounted for a 1.4% explanatory power regarding the variability (△R^2^ = 0.014). Obviously, peri-traumatic variables were the stronger predictors of hyper-arousal symptoms. Notably, the hyper-arousal symptoms of PTSD were observed to be elevated in patients marked by erratic daily routines (β = 0.441, p = 0.006) and attenuated in those exuding emotional serenity (β = −0.457, p = 0.004). It is imperative to highlight that the variables encompassed within the regression model provided only a fractional elucidation of the outcome, suggesting the possible influence of unexplored factors on the hyperarousal symptoms of PTSD, as visualized in Table 4 and Figure 1.

**Table 4:** Predictors of PTSD in hospitalized patients diagnosed with COVID-19 in Wuhan.

		Model 1				Model 2				Model 3		
Variable		Beta	*t*	*p*		Beta	*t*	*p*		Beta	*t*	*p*
Pre-traumatic information												
Gender (0 = Male, 1 = Female)		−0.002	−0.012	0.991		0.015	0.126	0.9		0.018	0.149	0.882
Age (0 = <60, 1 = ≥60)		−0.067	−0.467	0.642		−0.054	−0.401	0.69		−0.043	−0.321	0.75
Marital status (0 = Married, 1 = Unmarried)		−0.102	−0.801	0.426		−0.15	−1.27	0.21		−0.099	−0.806	0.424
Senior high school		−0.169	−0.93	0.356		−0.113	−0.706	0.483		−0.09	−0.564	0.575
Undergraduate		−0.147	−0.861	0.393		−0.09	−0.575	0.568		−0.018	−0.109	0.914
Post graduate and above		−0.032	−0.225	0.823		−0.052	−0.41	0.683		−0.049	−0.39	0.699
History of SARS infection (0 = No, 1 = Yes)		0.039	0.283	0.778		0.152	1.24	0.221		0.187	1.504	0.139
Pri-traumatic information												
Irregular daily routine (0 = No, 1 = Yes)						0.395	2.638	0.011		0.441	2.897	0.006
Disturbances in eating (0 = No, 1 = Yes)						−0.011	−0.094	0.925		−0.016	−0.134	0.894
Disturbances in sleeping (0 = No, 1 = Yes)						−0.09	−0.55	0.585		−0.091	−0.559	0.579
Illness degree (0 = Mild, 1 = Severe)						0.13	1.085	0.283		0.101	0.834	0.408
Serenity (0 = No, 1 = Yes)						−0.439	−2.873	0.006		−0.457	−3.006	0.004
Anxiety (0 = No, 1 = Yes)						0.056	0.387	0.701		0.044	0.306	0.761
Anger (0 = No, 1 = Yes)						−0.002	−0.018	0.986		−0.009	−0.073	0.942
Melancholy (0 = No, 1 = Yes)						−0.153	−0.972	0.336		−0.185	−1.173	0.246
Fear (0 = No, 1 = Yes)						0.258	1.787	0.08		0.249	1.74	0.088
Happiness (0 = No, 1 = Yes)						−0.179	−1.422	0.161		−0.216	−1.688	0.098
Total PTG score										0.176	1.374	0.175
*R* ^2^	−0.068				0.220				0.234			
△*R*^2^					0.288				0.014			
*F*									2.151			

### 3.5 Analysis of predictors of PTG in patients diagnosed with COVID-19 in Wuhan

**Figure 2:**
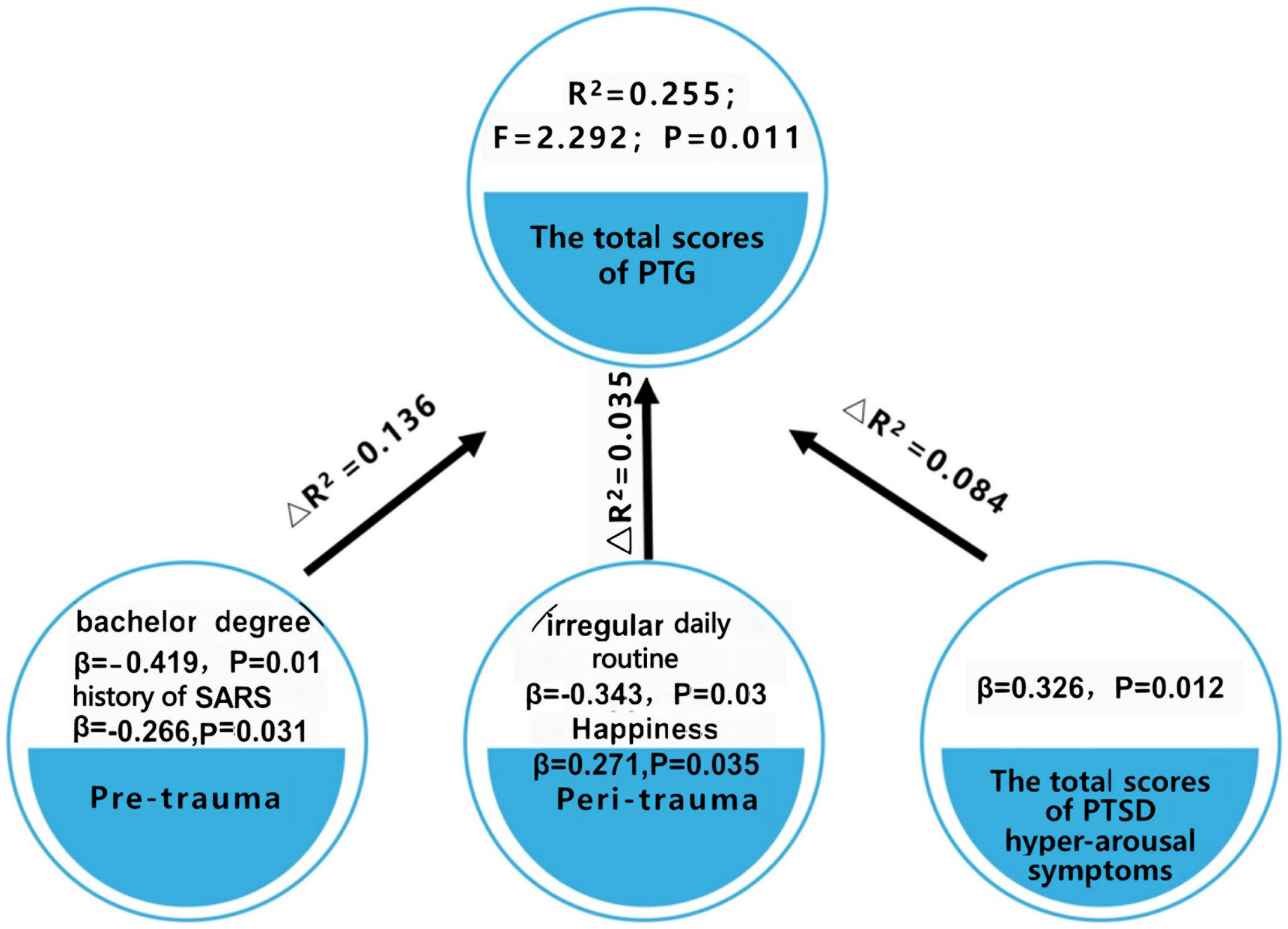
Evaluation of PTG predictors in patients diagnosed with COVID-19 in Wuhan.

A stratified regression analysis was conducted, stratified into three tiers, with the total score of PTG serving as the dependent variable (Table 5 and Figure 2). The factors perceived to potentially influence the PTG score were categorized into these three stratifications: The initial stratification focused on pre-trauma factors. The subsequent stratification incorporated factors pertinent during the trauma phase. The final stratification emphasized the positive rate of hyper-arousal symptoms of PTSD. Emerging from this analysis, five distinct variables—educational level, a history of SARS infection, irregular daily routines, a sensation of happiness, and the positive rate of hyper-arousal symptoms of PTSD were incorporated into the regression equation. In this context, the pre-trauma variables accounted for 13.6% of the variability in the PTG scores among patients diagnosed with COVID-19 in Wuhan (R^2^ = 0.136). The factors during the trauma phase explained 3.5% of the variability in the PTG scores (△R^2^ = 0.035). The positive rate of hyper-arousal symptoms of PTSD symptoms elucidated 8.4% of the variability in PTG (△R^2^ = 0.084).

**Table 5:** Predictors of PTG in hospitalized patients diagnosed with COVID-19 in Wuhan.

		Model 1				Moldel2				Model 3		
Variable		Beta	*t*	*p*		Beta	*t*	*p*		Beta	*t*	*p*
Pre-traumatic information												
Gender (0 = Male, 1 = Female)		−0.059	−0.503	0.617		−0.058	−0.458	0.649		−0.015	−0.128	0.899
Age (0 = 60, 1 = ≥60)		−0.042	−0.324	0.747		−0.154	−1.1	0.277		−0.15	−1.129	0.264
Marital status (0 = Married, 1 = Unmarried)		−0.111	−0.968	0.337		−0.237	−1.94	0.058		−0.223	−1.925	0.06
Senior high school		−0.079	−0.483	0.631		−0.16	−0.966	0.339		−0.094	−0.59	0.558
Undergraduate		−0.382	−2.493	0.015		−0.49	−3.024	0.004		−0.419	−2.687	0.01
Post graduate and above		0.05	0.398	0.692		−0.49	−3.024	0.004		0.071	0.571	0.57
History of SARS infection (0 = No, 1 = Yes)		−0.318	−2.578	0.012		−0.49	−3.024	0.004		−0.266	−2.218	0.031
Pri-traumatic information												
Irregular daily routine (0 = No, 1 = Yes)						−0.223	−1.443	0.155		−0.343	−2.234	0.03
Disturbances in eating (0 = No, 1 = Yes)						0.022	0.176	0.861		0.033	0.277	0.783
Disturbances in sleeping (0 = No, 1 = Yes)						−0.015	−0.087	0.931		0.036	0.225	0.823
Illness degree (0 = Mild, 1 = Severe)						0.088	0.715	0.478		0.11	0.938	0.353
Serenity (0 = No, 1 = Yes)						−0.07	−0.443	0.66		0.035	0.226	0.822
Anxiety (0 = No, 1 = Yes)						−0.253	−1.698	0.096		−0.257	−1.819	0.075
Anger (0 = No, 1 = Yes)						−0.113	−0.901	0.372		−0.154	−1.29	0.203
Melancholy (0 = No, 1 = Yes)						−0.02	−0.123	0.902		−0.015	−0.099	0.921
Fear (0 = No, 1 = Yes)						0.29	1.948	0.057		0.235	1.647	0.106
Happiness (0 = No, 1 = Yes)						0.217	1.668	0.102		0.271	2.171	0.035
Hyper-arousal symptoms of PTSD (0 = Negative, 1 = Positive)										0.326	2.602	0.012
*R* ^2^	0.136				0.171				0.255			
△*R*^2^					0.035				0.084			
*F*									2.292			

Notably, higher PTG scores were observed in patients exuding happiness (β = 0.271, p = 0.035) and those testing positive for hyperarousal symptoms of PTSD (β = 0.326, p = 0.012). In contrast, patients with a bachelor’s degree (compared to those with elementary schooling) (β = −0.419, p = 0.01), those with a history of SARS (β = −0.266, p = 0.031), and those with irregular daily routine (β = −0.343, p = 0.03) registered diminished PTG scores.

It is pertinent to underscore that the variables incorporated within this regression model elucidated only a fraction of the overall outcome. This suggests the probable influence of additional factors, which remain unexplored in this study, on the PTG scores, as depicted in Table 5 and Figure 2.

## 4 Discussion

This study is one of the first to report the prevalence of hyper-arousal symptoms and PTG in inpatients with COVID-19 at Wuhan during their early days of hospitalization. These inpatients experienced strong psychological turbulence during the early hospitalization stage; i.e., more stress and more growth. The primary physiological symptoms pinpointed in these patients were an inconsistent daily routine and sleep disturbances. Both these manifestations were more pronounced than in healthy individuals during the same timeframe. On the emotional spectrum, a significant majority, 69.6%, exuded serenity, while negative emotions like anxiety, anger, melancholy, and fear were reported at lower rates (anxiety, 18.6%; anger, 2%; melancholy, 10.8%; and fear, 8.8%). The study conducted by Wang, which assessed suicidal tendencies, sleep conditions, and psychological health of COVID-19 patients, suggested an anxiety rate of 31.91%, a figure that diverges from our findings (Wang et al., 2020). The finding indicates that inpatients experience a certain level of psychosocial responses at the early stage of hospitalization when doctors are likely to focus on physical treatment.

In the face of the COVID-19 pandemic, a major stress-inducing event, psychological crises of varying intensities have been observed across individuals and communities. Post-infection, a considerable number of patients exhibited heightened alertness, characterized by diminished concentration, disrupted sleep patterns, irritability, increased sensitivity, and increased startle response. Our study found that 37.3% of the hospitalized patients in Wuhan displayed hyperarousal symptoms of PTSD, surpassing the 13.1% in healthy counterparts during the same period. Similarly, a previous study investigated the psychiatric symptoms in patients who recovered from COVID-19 and reported that 25.4% of patients reported clinical PTSD (Poyraz et al., 2021). One probable explanation is that our study concentrated on the early hospitalization stage, which may be characterized by more psychological trauma due to unfamiliar isolation treatment. In addition, during the first pandemic peak of the COVID-19 outbreak in Wuhan, during which the novel coronavirus was of unknown origin, aggressively contagious, lacked effective diagnostic and therapeutic protocols, and had a high morbidity and mortality rate. As a result, patients may have had a more intense fear of the acute hazards posed by the unknown virus, which could also have affected their psychological well-being. The most pressing symptom identified was disrupted sleep, accentuating the importance for healthcare practitioners to focus on sleep regulations for mitigating PTSD levels.

Defined by Tedeschi and Calhoun as the positive psychological metamorphosis post trauma, PTG was observed in 77.5% of the COVID-19 patients in our study (Tedeschi and Calhoun, 1996). This surpassed the scores in the healthy demographic for the same timeframe. A PTG rate of 51.1% was previously reported among earthquake survivors (Jin et al., 2014). Additionally, Calhoun and Tedeschi (Calhoun and Tedeschi, 2014) concluded in a review that the prevalence of PTG in individuals ranged from 30 to 90%, which covers our findings. This elevation in PTG post the traumatic experience of the pandemic can be attributed to the ability of the patients to introspect, gain a renewed perspective on life, bolster personal resilience, and consequently attain higher PTG levels. Further, the most notable score was observed in interpersonal relationships, in line with the research conducted by Dong (Dong, 2016). One possible explanation is that during the patient’s hospitalization and isolation, the patient is able to receive specialized care and assistance from healthcare professionals, which increases trust and dependence on others, ushering in greater emotional expression and proactive help-seeking behavior.

The results suggested that patients with COVID-19 in Wuhan had a more significant psychological turbulence during the early stage than the healthy demographic. Indeed, higher hyper-arousal and growth in patients showed that the patients had a rough time during the early hospitalization stage. However, it should be noted that, in the early stage of the COVID-19 outbreak, much emphasis was placed on the biological, epidemiological, and clinical characteristics of COVID-19, whereas limited research has addressed the psychological consequences among COVID-19 patients. The findings motivated healthcare workers to pay attention to trauma and growth responses in patients simultaneously, i.e., reducing psychological trauma while promoting psychological growth in interventions.

Utilizing the three-phase trauma theory, we analyzed the influence of various trauma phases on PTSD and PTG in patients. The results revealed that pre-trauma factors were less predictive of PTSD than those during trauma, this is consistent with previous findings that pre-traumatic factors are the weakest predictors of PTSD compared to pri-and post-traumatic factors. As inferred by the meta-analysis on children and adolescents conducted by Trickey, pre-trauma factors might only be indicative of PTSD in specific subsets, and their predictability can vary across different cohorts (Trickey et al., 2012). This assertion warrants further research with more extensive sample sizes. In terms of PTG, both pre-trauma and pri-trauma variables held predictive value, whereas educational background and history of SARS exposure, as two pre-traumatic variables, are important factors influencing PTG.

Previous studies demonstrated that the subjective emotional response of a patient during trauma serves as a pivotal predictor of both PTSD and PTG (Carlier et al., 1997). Consistent with this paradigm, the results of our study reaffirm the significance of this relationship. In particular, the experience of serenity and happiness during trauma—both signifiers of affirmative emotional responses—stand as robust protective factors against hyper-arousal symptoms of PTSD and concurrently bolster PTG. A salient observation is that hospitalized patients who retain feelings of serenity and happiness manifest lower scores pertaining to hyper-arousal symptoms of PTSD and higher scores for PTG. However, few studies investigated subjective emotional reactions throughout the pri-traumatic period, and most of them focused on negative feelings such as worry and terror (Chen et al., 2021). Our study comprehensively analyzed the prediction of emotional responses on patients’ psychological well-being from the perspective of both positive and negative emotion, which is the first report of its kind. Though our findings could not validate adverse emotional responses of patients during trauma as predictive factors for hyper-arousal symptoms of PTSD and PTG, extant studies postulate that resilience-promoting factors can potentially illuminate corresponding risk factors (Iacoviello Brian and Charney, 2014). Hence, we hypothesize that the adverse emotional response of patients during trauma might predict risks associated with hyper-arousal symptoms of PTSD and PTG, albeit this necessitates further empirical substantiation. Further, we found that an irregular sleep pattern during hospitalization emerged as a risk factor for hyper-arousal symptoms of PTSD and PTG. Those with inconsistent sleep manifested elevated hyper-arousal symptoms of PTSD and diminished PTG scores. Sun (Sun et al., 2021) revealed a significant correlation between sleep quality and PTSD symptom levels in COVID-19 patients in his study, which is consistent with our results. Sleep disturbances were common among people involved in the COVID-19 pandemic (Liu et al., 2020; Sun et al., 2021). Compared with PTSD, sleep disturbances are more likely to be recognized by clinicians. Timely identification and treatment for trauma-induced sleep problems may prevent the development of PTSD.

A noteworthy dimension of our findings was the realization that, concerning both hyper-arousal symptoms of PTSD and PTG, the identified variables in the regression analysis illuminated only a fragment of the model outcomes. This insinuates the likelihood of unaccounted factors influencing the psychological well-being of hospitalized patients diagnosed with COVID-19 in Wuhan. The focal point of our inquiry was the hospitalization period, primarily assessing pre-trauma and during-trauma factors. Prior studies emphasize the predictive potential of post-traumatic psychosocial factors, such as supportive social networks, optimism, cognitive adaptability, and affirmative beliefs, which potentially align with our findings and place emphasis on post-traumatic factors (Sayed et al., 2015).

With PTSD and PTG epitomizing the dualistic outcomes post trauma—reflecting both detrimental impacts and positive growth trajectories—our findings infer that hyper-arousal symptoms of PTSD may be essential for PTG. Evidently, patients manifesting hyper-arousal symptoms of PTSD tend to register elevated PTG scores. This mirrors findings from previous studies of COVID-19 pandemic populations (Bonazza et al., 2022; Collazo-Castiñeira et al., 2022). According to previous reports, post-traumatic development (spiritual transformation, appreciation of life, new possibilities, and personal strength) was positively associated with post-traumatic stress. The conjecture is that post traumatic occurrences, individuals may encounter heightened alertness reactions, inducing self-rumination and introspection, catalyzing core belief reformation, culminating in PTG (Liu et al., 2017). Thus, while trauma may destabilize and challenge core beliefs, the ensuing dissonance, although disconcerting, could be a catalyst for growth.

## 5 Implications

This cross-sectional study examines the psychological distress experienced by early hospitalized patients during the first peak of the COVID-19 outbreak in Wuhan. The study is valuable for understanding the impact of the pandemic on patients’ mental health. The study found that certain variables during the trauma period, specifically irregular daily routines and positive perception of emotions, were strong predictors of hyper-arousal symptoms and post-traumatic growth (PTG). These variables were even stronger predictors than demographic variables, which are typically stable and immutable. This suggests that patients’ irregular daily routines and subjective perception of emotions during hospitalization have a significant impact on their psychological well-being. Is it possible to intervene early in a patient’s hospitalization to prevent PTSD after discharge and promote post-traumatic growth? Firstly, to improve patients’ positive emotions, health workers can help them adapt in three ways: (1) health workers should help patients realize that it is normal to experience negative emotions when going through a serious traumatic event. (2) Patients should be encouraged to adopt positive coping styles, such as communicating with family or friends or engaging in activities that interest and give pleasure to them. (3) Optional and optimal digital mental health services should be provided. For instance, an online counseling platform can facilitate hospitalized patients to seek help independently and relieve psychological stress. Secondly, to adjust the irregular daily routine of patients, the following measures can be taken: (1) Improving the ward environment by reducing noise levels, such as talking and alarms, and softening the lighting. (2) Providing patients with noise-canceling earplugs and light-blocking eye masks when they need to rest. (3) Hospitalized patients can benefit from a range of behavioral interventions, such as music therapy (Majid et al., 2023), mindfulness meditation (Johnson Leslie et al., 2023), and the most evidence-based treatment might be cognitive behavior therapy (CBT) (Redeker Nancy, 2023), especially the Internet CBT that can prevent the spread of infection during the pandemic.

## 6 Limitations of this study

This study, while providing valuable insights, is not without its limitations. First, a primary constraint pertains to the exclusive focus on pre-trauma and pri-trauma factors in assessing the psychological health of patients. However, the results of our study allude to the potential significance of post-traumatic factors in influencing the psychological health of patients, thus carving a pathway for prospective research endeavors. Second, our sampling methodology excluded patients who faced challenges in utilizing communication tools or needed external assistance to navigate the online questionnaire. This potentially marginalizes a segment of the population, notably those with limited educational backgrounds. Given that education level surfaces as a potentially influential factors of psychological health, this exclusion could skew our findings. Future investigations would benefit from adopting a more inclusive approach, ensuring a holistic representation of the target demographic. Third, potential bias might exist in this study, such as selection bias in the recruitment of participants (the extremely severe patients were excluded whose psychological responses might be worse) and information bias in the participants’ psychological condition prior to admission (participants’ baseline psychological condition might influence their psychological responses during hospitalization). To reduce or avoid potential bias, clinical nurses and psychiatrists worked together to communicate with participants and collect information during the period of study design and conduction. Furthermore, data quality control was practiced rigidly during the whole process of study.

## 7 Conclusion

This research stands out due to its pioneering approach in coalescing PTSD and PTG, presenting them as intertwined facets of the post-traumatic psychological spectrum, and methodically delineating the psychological landscape of patients diagnosed with COVID-19 during their hospitalization in Wuhan. Drawing upon the pre-trauma, pri-trauma, and post-trauma conceptual framework, this study offers a comparative analysis of the predictive powers of factors across these trauma phases on the psychological health of patients. Of particular note is our exploration into the influence of subjective emotional experiences during trauma, framing it as a predictor for both PTSD and PTG from a constructively oriented viewpoint. This augments our comprehension of the intricate psychological responses that patients manifest in the wake of traumatic episodes. Such insights not only enrich the academic discourse around trauma-induced psychological responses but also establish a robust empirical foundation for crafting nuanced, enduring, and systematic therapeutic interventions tailored for the psychological trauma endured by patients diagnosed with COVID-19.

## Data availability statement

The original contributions presented in the study are included in the article/supplementary material, further inquiries can be directed to the corresponding author.

## Ethics statement

The studies involving humans were approved by the Ethics Committee of Army Medical University. The studies were conducted in accordance with the local legislation and institutional requirements. The participants provided their written informed consent to participate in this study.

## Author contributions

J-jC: Conceptualization, Resources, Writing – original draft, Writing – review & editing. BY: Data curation, Formal analysis, Writing – original draft. LY: Data curation, Formal analysis, Writing – original draft. X-xS: Data curation, Formal analysis, Writing – review & editing. QD: Conceptualization, Methodology, Writing – review & editing.

## Footnotes

^1^ https://www.wjx.cn
